# Volatile Phenols—Important Contributors to the Aroma of Plant-Derived Foods

**DOI:** 10.3390/molecules25194529

**Published:** 2020-10-02

**Authors:** Andreas Schieber, Matthias Wüst

**Affiliations:** 1 Chair of Molecular Food Technology, Institute of Nutritional and Food Sciences, University of Bonn, Endenicher Allee 19B, 53115 Bonn, Germany; schieber@uni-bonn.de; 2 Chair of Food Chemistry, Institute of Nutritional and Food Sciences, University of Bonn, Endenicher Allee 19B, 53115 Bonn, Germany

**Keywords:** polyphenols, glycosyltransferase, odor perception, biosynthesis, olfaction, key food odorants

## Abstract

Volatile phenols like phenylpropanoid and benzoid compounds originate from the aromatic amino acid phenylalanine, which is biosynthesized via the shikimate/arogenate pathway. These volatile compounds contribute to the aroma of a number of economically important plant-derived foods like herbs, spices and fruits. The sequestration of numerous phenylpropanoid and benzoid compounds as glycosides occurs widely in fruits, and this pool represents an important source of flavor that can be released during storage and processing. Therefore, this review will provide an overview of the biosynthesis of free and glycosylated phenylpropanoid and benzoid compounds and their reactions during food processing, which both lead to the generation of odor-active volatile phenols in plant-derived foods.

## 1. Introduction

Intensive research during the last two decades has shown that flavonoids, stilbenes, tannins, phenylpropanoids, lignans and coumarins (often generically called (poly)phenols to illustrate that this class of compounds possess one or multiple aromatic rings and at least one hydroxyl group) are important secondary metabolites in plant-derived foods. Over 7000 known structures have been reported so far, illustrating the high structural diversity of these natural products [1]. The pronounced dynamics in cross-disciplinary research on (poly)phenols are mainly due to their putative health benefits on the one side and their role as technologically valuable natural food ingredients on the other side; the latter intended to replace synthetic food additives. For example, polyphenols have been associated with positive effects on cardiometabolic health, cognition, type II diabetes, obesity, neuroinflammation and others [2,3,4,5]. More recent research has demonstrated that a significant part of the polyphenols ingested is metabolized by human endogenous enzymes and the gut microbiota, and increasing evidence indicates that the metabolites, rather than the parent polyphenols present in the plant, constitute the bioactive compounds [6,7,8]. From a technological point of view, polyphenols are of interest because they contribute to color [9], antioxidant activity [10] and preservation of foods [11]. In addition, some phenolic compounds, such as caftaric acid and tannins, are known to impart bitterness and astringency [12,13,14]. However, (poly)phenols can also shape the aroma of foods if they are volatile as such or serve as precursors for volatile degradation products. This review is therefore focused on highly odiferous volatile phenols that contribute to the aroma of foods, either as positive contributors or as so-called off-flavor compounds in tainted products of diminished quality.

## 2. The Concept of the Chemical Odorant Space

Food contains over 10,000 volatiles with molecular weights below 290 Da [15]. However, while volatility is a prerequisite for a compound to be perceived nasally, only a small fraction of these molecules contributes to the overall aroma of food. So far, about 230 volatiles have been identified as so-called “key food odorants” (KFOs) [16]. These KFOs are the best natural agonists for approximately 400 human olfactory receptors (ORs) [17]. Aroma research has shown that the distinctive aroma for 227 different food commodities is determined by a relatively small subset of 3 to 40 KFOs. For example, the distinct butter aroma consists of only three compounds: diacetyl, (*R*)-*δ*-lactone and butyric acid. These three compounds are present in butter at concentrations above their odor threshold values and represent the chemical odor code for a buttery aroma. The 230 KFOs span, therefore, the chemical odorant space of our food.

## 3. Odor-Active Phenols in Food

**Figure 1:**
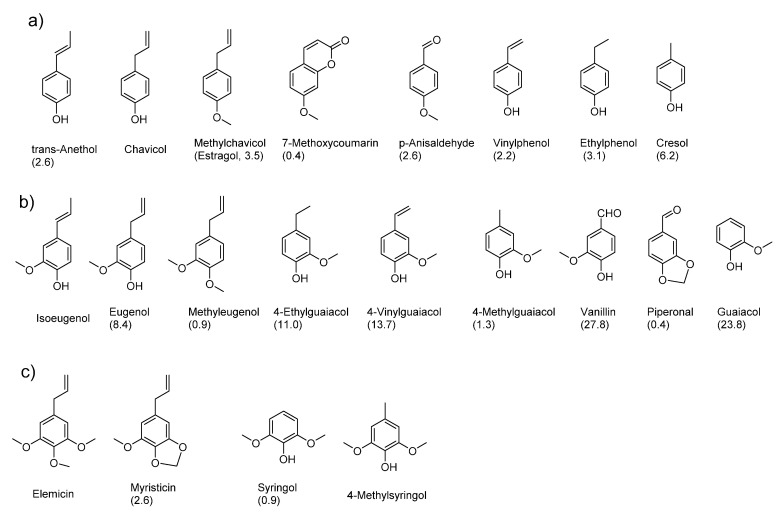
Structures of volatile odor-active phenols that were detected in 227 food samples [16]. Below each structure the trivial name and, in parentheses, the frequency of occurrence in percent in the 227 food samples are given. Mono- (**a**), di- (**b**) and trihydroxylated (**c**) derivatives are shown.

About 10% of all known KFOs are volatile phenols, and their structures are listed in Figure 1, together with their trivial names and, in parentheses, their frequency of occurrence in percent in 227 food samples.

The top three odor-active phenols in food are vanillin, guaiacol and 4-vinylguaiacol. In spices, herbs and fruits, phenols with a C_3_ sidechain are found frequently, whereas in fermented food like alcoholic beverages, the C_3_ sidechain is often shortened to one or two C atoms by microbial degradation (see below). The aromatic ring can bear up to three hydroxyl groups. However, no more than one hydroxyl group is present in free form in these structures, and polyphenolic molecules are missing completely because of their greatly reduced volatility, which excludes any aroma activity.

## 4. Perception of Odor-Active Phenols

The biochemical mechanism for perception of odor-active phenols is now well understood and is a remarkable piece of Nobel Prize winning research (Nobel Prize in Physiology or Medicine 2004 for Richard Axel and Lina B. Buck for their discoveries of odorant receptors and the organization of the olfactory system) [17]. Odor perception is initiated by an odorant molecule binding to receptors that are located in the cilia of olfactory receptor cells in the nasal epithelium. The cells are thus activated and send electric signals that are relayed in the so-called glomeruli in the olfactory bulb. The signals are transmitted to higher regions of the brain, and the integrated signal pattern is finally interpreted as a specific odor impression. It is clear that these olfactory receptors (ORs) play a key role in odorant perception. Up to now, for approximately 40% of the 400 functional human ORs, agonists have been identified in bioassays [18]. Research on structure–activity relationships of the human OR10G receptors has shown that these receptors are broadly tuned to volatile phenols (Table 1) [19]. Obviously, the phenolic foodborne stimulus space has co-evolved with our olfactory receptors. It is utmost remarkable that these nasal receptor proteins are also expressed in other tissues like the skin, the intestine and even in white blood cells. Thus, it is conceivable that these small molecules exert other biological effects on the human body beside their odor activity, and once ingested they may overcome cellular barriers and reach other target tissues. Recent research on low-molecular weight metabolites from polyphenols as effectors for attenuating neuroinflammation and for prevention of cardiovascular diseases shows that this rationale is, indeed, valid [4,6].

## 5. Generation of Volatile Phenols in Food

### 5.1. De Novo Biosynthesis in Planta

**Figure 2:**
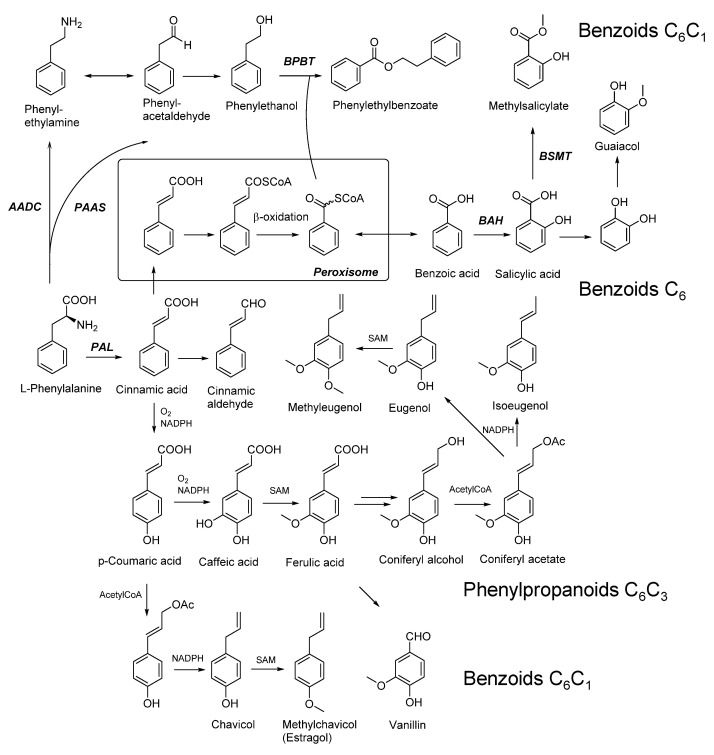
Metabolic pathways in plants that lead to the formation of volatile benzoids and phenylpropanoids from phenylalanine [31]. AADC: aromatic amino acid decarboxylase; BAH: benzoic acid 2-hydroxlase; BPB: benzoylCoA:benzylalcohol/2-phenylethanol benzoyltransferase; BSMT: benzoic acid/salicylic acid carboxyl methyltransferase; CoA: coenzyme A; NADPH: nicotinamide adenine dinucleotide phosphate (reduced form); PAAS: phenylacetaldehyd synthase; PAL: phenylalanine ammonia lyase; SAM: S-adenosyl methionine.

In planta, volatile phenols are generated from phenylalanine (Figure 2) that delivers the C_6_C_3_ building block of all phenylpropanoids [20,21]. The key enzyme is the well-characterized enzyme phenylalanine ammonia lyase (PAL) that controls the flux through this pathway [22]. The pathway, up to the production of coumaryl and coniferyl alcohol, is shared with the lignin biosynthetic pathway. Hydroxylation of the aromatic ring and subsequent reduction of the corresponding acetate ester yields volatile phenylpropanoids of the widespread C_6_C_3_-type. The enzymes responsible for acetate ester formation and reduction have been characterized in sweet basil and anise [23,24,25]. The genes involved in the biosynthesis of methoxylated phenylpropenes have recently been cloned and characterized, notably in strawberry, apple, tomato and grape [20]. For example, manipulating the levels of phenylpropenes validated the importance of eugenol to strawberry fruit aroma [26,27], thus demonstrating how this knowledge can ultimately lead to the improvement of crops with respect to their odor traits. Chain-shortening by β-oxidation finally yields C_6_C_1_- and C_6_-type benzoids. A special case is the generation of vanillin from ferulic acid, which is catalyzed by the action of a single enzyme that has recently been cloned and characterized from *Vanilla planifolia* [28,29]. Likewise, a gene for *p*-hydroxystyrene biosynthesis has been characterized in *Pyrus communis* that encodes a phenolic acid decarboxylase yielding *p*-hydroxystyrene from *p*-coumaric acid [30].

### 5.2. Microbial Formation of Volatile Phenols

**Figure 3:**
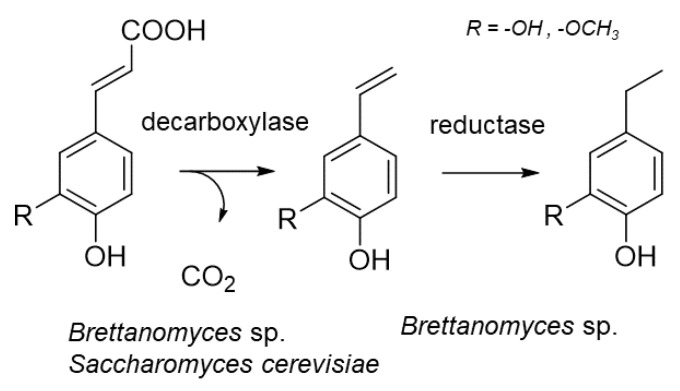
Formation of allyl- and ethylphenols by yeast during fermentation.

During food processing by fermentation, microorganisms are capable of degrading nonvolatile phenylpropanoids to volatile, highly odiferous phenols. Showcased is the yeast-mediated formation of ethyl- and allylphenols by decarboxylation and reduction during the alcoholic fermentation in wine and beer production (Figure 3) [32]. If these degradation products are formed in excessively high concentrations, aroma defects, like a “horse sweat” note in wine, can develop [33,34].

**Figure 4:**
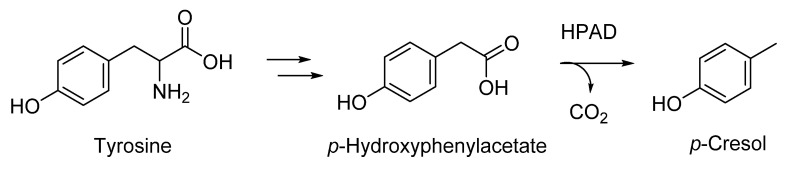
Formation of *p*-cresol by anaerobic-fermenting bacteria. HPAD: *p*-hydroxyphenylacetate decarboxylase.

Degradation of aromatic amino acids, such as tyrosine, during fermentation can yield volatile phenols as well. The cresol-forming enzyme *p*-hydroxyphenylacetate decarboxylase (HPAD) is a member of the glycyl radical enzyme (GRE) superfamily and plays prominent roles in the primary metabolism of anaerobic-fermenting bacteria (Figure 4) [35].

The catalytic mechanism of HPAD has been studied in detail and involves activation of p-hydroxyphenylacetate (HPA) by concerted abstraction of an electron and the phenolic proton to generate a phenoxy-acetate radical anion, with the radical delocalized over the aromatic ring [36]. Decarboxylation and H-radical rebound finally yields *p*-cresol, which is released from the enzyme by exchange with HPA.

### 5.3. Thermal Formation of Volatile Phenols During Food Processing

**Figure 5:**
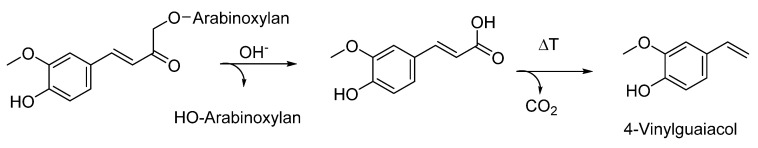
Formation of 4-vinylguaiacol from arabinoxylans during soft pretzel production [38].

Volatile phenols can be formed from labile precursors during thermal treatment of food [37]. In soft pretzels, 4-vinylguaiacol can be generated from hemicelluloses like arabinoxylans that contain covalently bound ferulic acid, which is liberated during the alkaline treatment of the dough and thermally decarboxylated by the subsequent baking process (Figure 5) [38]. During coffee roasting, decarboxylation of ferulic acid yields 4-vinylguaiacol, guaiacol and phenol [39]. The pyrolysis of wood yields a variety of volatile phenols as well that are responsible for the antioxidative and aromatic properties of smoke, which is used for food conservation by smoking [40] and wine aromatization by storage in toasted barrels [41,42].

## 6. Glycosidically Bound Volatile Phenols in Food

### 6.1. Structures of Glycosidically Bound Phenols

**Figure 6:**
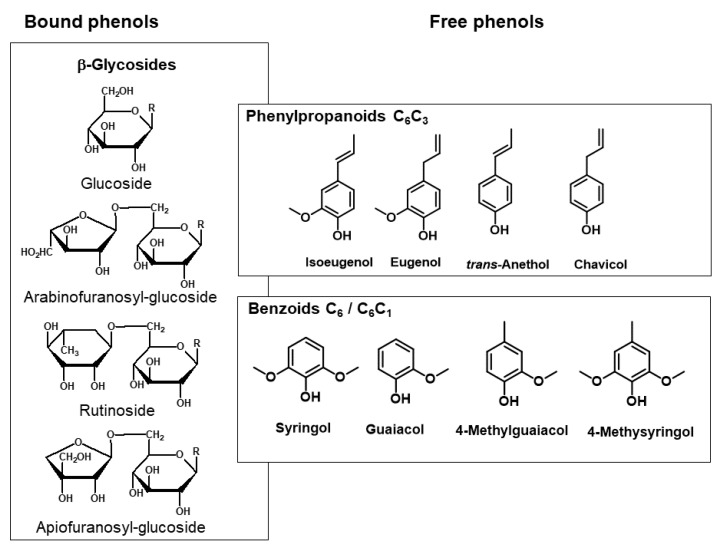
Structures of glycosidically bound volatile phenols.

Sequestration of phenols as glycosides occurs commonly in fruit, and this so-called bound pool represents an important source of flavor compounds that can be released during processing, as well as by controlled application of enzymes, heat or acids [43]. Mono- and diglycosides like glucosides, arabinofuranosylglucosides, rutinosides and apiofuranosylglucosides are structures frequently occurring in fruits (Figure 6) [31].

### 6.2. Biosynthesis of Glycosidically Bound Phenols

**Figure 7:**
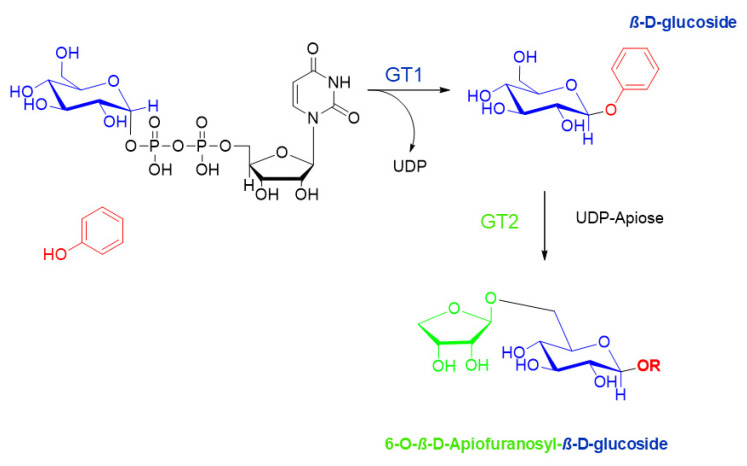
Sequential biosynthesis of glycosides by uridine diphosphate-glycosyltransferases (UDP-GTs).

In general, the biosynthesis of glycosidically bound small molecule volatiles is catalyzed by uridine diphosphate glycosyltransferases (UDP-GTs) [44,45]. These enzymes use UDP-activated sugars as cofactors and transfer the sugar moiety onto the aglycone by an S_N_2-type reaction with inversion of the configuration at the anomeric C-atom, resulting in the formation of β-glycosides. By sequential biosynthesis, further sugar molecules can be transferred onto the first one by forming diglycosides like apiofuranosyl-glucosides (Figure 7). All plant UDP-GTs show a typical GT-B fold-structure and harbor a highly conserved plant secondary product glycosyltransferase (PSPG) motif that enables UDP-sugar donor binding at the active site. Cloning, heterologous expression and biochemical characterization of UDP-glucose: small-molecule GTs from *Vitis vinifera* have shown that these enzymes show acceptor promiscuity and glycosylate structurally diverse substrates like monoterpenes, (poly)phenols and aliphatic alcohols [46,47,48].

However, this promiscuity can be the cause of smoky off-flavor notes in wines [49]. Volatile phenols that are generated during bush fires can penetrate into the leaf tissue of grapevine. It was shown that these phenols can be glycosylated by promiscuous GTs, translocated into the berry and liberated during storage of the produced wine, thus tainting this product with a smoky off-flavor [50,51,52].

## 7. Conclusions

This review has provided an overview of the biosynthesis of free and glycosylated phenylpropanoid and benzoid compounds and their reactions during food processing, with an emphasis on the odor-active phenols and their glycosylated precursors. Molecular sensory science and the concept of the chemical odorant space have impressively shown that these volatile phenols are indeed important contributors to the aroma of plant-derived foods. Knowledge on the biosynthesis of these target molecules, including the underlying regulation mechanisms, will ultimately lead to the improvement of crops with respect to their odor traits [53].

## Figures and Tables

**Table 1:** Structure–activity relationship of OR10G receptors [19].

Structure	OR10G3	OR10G4	OR10G7	OR10G9
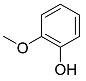	^1^++	+++	++	++
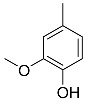	++	++	++	++
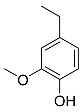			++++	
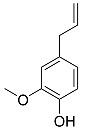	+		++++	
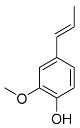	++	++	++++	+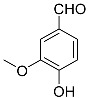	++	++	+	+

^1^ +: EC50 > 1 mM; ++: EC50 < 1 mM; +++: EC50 < 100 µM; ++++: EC50 < 10 µM.
